# New generation of drug delivery systems based on ginsenoside Rh2-, Lysine- and Arginine-treated highly porous graphene for improving anticancer activity

**DOI:** 10.1038/s41598-017-18938-y

**Published:** 2018-01-12

**Authors:** Hadi Zare-Zardini, Asghar Taheri-Kafrani, Ahmad Amiri, Abdol-Khalegh Bordbar

**Affiliations:** 1 Department of Biotechnology, Faculty of Advanced Sciences and Technologies, University of Isfahan, Isfahan, 81746-73441 Iran; 2 Department of Mechanical Engineering, Faculty of Engineering, University of Malaya, 50603 Kuala Lumpur, Malaysia; 3 Department of Chemistry, University of Isfahan, Isfahan, 81746-73441 Iran

## Abstract

In this study, Rh2–treated graphene oxide (GO-Rh2), lysine-treated highly porous graphene (Gr-Lys), arginine-treated Gr (Gr-Arg), Rh2–treated Gr-Lys (Gr-Lys-Rh2) and Rh2–treated Gr-Arg (Gr-Arg-Rh2) were synthesized. MTT assay was used for evaluation of cytotoxicity of samples on ovarian cancer (OVCAR3), breast cancer (MDA-MB), Human melanoma (A375) and human mesenchymal stem cells (MSCs) cell lines. The percentage of apoptotic cells was determined by terminal deoxynucleotidyl transferase-mediated dUTP nick-end labeling (TUNEL) assay. The hemolysis and blood coagulation activity of nanostructures were performed. Interestingly, Gr-Arg, Gr-Lys, Gr-Arg-Rh_2_, and Gr-Lys-Rh_2_ were more active against cancer cell lines in comparison with their cytotoxic activity against normal cell lines (MSCs) with IC_50_ values higher than 100 μg/ml. The results of TUNEL assay indicates a significant increase in the rates of TUNEL positive cells by increasing the concentrations of nanomaterials. Results were also shown that aggregation and changes of RBCs morphology were occurred in the presence of GO, GO-Rh_2_, Gr-Arg, Gr-Lys, Gr-Arg-Rh_2_, and Gr-Lys-Rh_2_. Note that all the samples had effect on blood coagulation system, especially on PTT. All nanostrucure act as antitumor drug so that binding of drugs to a nostructures is irresolvable and the whole structure enter to the cell as a drug.

## Introduction

Ginsenoside Rh2 (Rh2) is a new anticancer natural agent extracted from ginsenosides. This is a triterpene glycoside saponin. It belongs to the family of 20 (S)- protopanaxadiol saponins^1,2^. Different studies showed that this compound could inhibit the growth of some kinds of cancer cells such as breast cancer^3,4^, prostate cancer^5^, pancreatic cancer^2^, gastric cancer^6^, hepatocellular carcinoma^7^, skin squamous cell carcinoma^8^, and another carcinoma^9,10^. Different mechanisms were proposed for anticancer activity of Rh2 e.g., regulation of cancerous cell by protein signaling, change of telomerase activity, inhibition of cell metabolism, the sensation of tumor cell to apoptosis, etc.^5,9–12^. Besides all the promising properties of Rh2, there is a fundamental challenge for realizing the application of Rh2: low oral bioavailability because of its hydrophobic property and fast plasma elimination by glycosidase^13,14^. Therefore, utilization of pure Rh2 molecules without any substrates as adjuvant lead to its elimination from the gastrointestinal tract or blood circulation before reaching to the target cells. So, important challenges for employing Rh2 as an anticancer drug are *in vivo* residence in the gastrointestinal tract and blood circulation. There are some strategies for these purposes. A few studies reported some drug delivery systems by using natural or synthetic vehicles such as liposome and multicore noisome for enhancement of therapeutic indices of Rh2^15–17^. Application of liposome and multicore noisome as drug delivery system is limited by liposomal aggregation and drug leakage. These problems affected the quality of liposomal systems^17^. In between, nanotechnology and its related compounds have very suitable advantages for application as drug targeting systems. Recently, it has been reported that nanotechnology is more efficient in the enhancement of drug delivery to different tissues^18^. Due to promising properties of carbon-based nanostructures such as high specific surface area, they can be suitable delivery vehicles for targeted delivery of Rh2 as well as protection of Rh2 from oral digestion and enzymatic lysis by glycosidase. Recently, nanostructures have been considered as a new drug delivery system for treatment of cancer. To this end, anticancer drugs are encapsulated in ordered nanostructures and delivered to cancerous cells^19–21^. As another positive point, different nanostructures such as metal nanoparticles, nanotubes, etc. have potent anticancer activity^22–24^. The main aims of conjugation of drugs with nanostructures are enhancement of activity on cancerous cells as well as unstable drug retention in patient’s body and reduction of side effect on normal cells for both drugs and nanostructures^25–28^. Among different nanomaterials, graphene has been highly regarded by different scientists as a potent drug delivery substrate^29–32^. It is a single, tightly packed layer of carbon atoms that are bonded together in a hexagonal honeycomb lattice. Due to unique properties, graphene sheets are also used in various medical, chemical and industrial processes. The modification or functionalization of graphene by various functional groups can improve its activity^33–36^. In previous studies, it has been proved that functionalization of CNTs by acidic amino acids enhance their biological activity. Functionalization by these amino acids leads to more positively charged surfaces^37–39^. The similar strategy can be used for graphene. Our first hypothesis is that functionalization with amino acids can enhance surface charge and facilitate directed entrance into the cancerous cell because of high negative charge of cancer cells. Our second hypothesis is that the addition of Rh2 to positively-charged systems with the very high specific surface area can protect its backbone for delivery to the cancer cells. Graphene with the high specific surface area can be a suitable substrate for high loading of Rh2. Additionally, because of anticancer activities of graphene, Rh2-based graphene can increase antitumor activity. Therefore, in this study, several Rh2-treated graphene-based nanosystems were synthesized for improving the anticancer performance of Rh2 and reduction of blood and oral elimination.

## Materials and Methods

### Materials

Ginsenoside Rh2, RPMI-1460 and L-glutamine were purchased from Sigma, and Antibiotic/Antimycotic Solution and fetal bovine serum were purchased from Gibco (Grand Island, NY). 3-(4,5-dimethylthiazol-2-yl)-2,5-diphenyltetrazolium bromide (MTT) was purchased from Roche (Applied Science, Indianapolis, IN). Arginine, lysine, dimethylacetamide (DMA) and sodium nitrite (NaNO2), all with analytical grade were obtained from Merck Inc. All other chemicals used were of analytical grade.

### Preparation of graphene oxide-ginsenoside Rh2 (GO-Rh2)

An easy modified Hummer’s method was developed for the preparation of graphene oxide (GO) from graphite powder^40^. Typically, 2 g of graphite powder and 2 g of NaNO_3_ were added in conc. H_2_SO_4_ (50 mL) at 0 °C. While maintaining vigorous stirring, KMnO_4_ (4.0 g) was added slowly to the mixture. The color of the suspension turned into purple-green immediately. The mixture was stirred at 40 °C until it became pasty brownish. The brown colored paste was diluted with 50 mL of deionized water (DI) and allowed to stir for 10 min. Then, 10 mL of H_2_O_2_ (30% wt.) solution was slowly added to the mixture, after which the color of the mixture changed to golden-brown. The golden-brown sol acquired in this step was the nanocomposites of graphene oxide. This mixture was centrifuged to collect the bottom product and sequentially washed with HCl solution (1 M) and warm DI water for several times to remove the residual metal ions. The powder was dried at room temperature, under vacuum condition.

For the preparation of graphene oxide-ginsenoside Rh2 (GO-Rh2), in a typical experiment, 10 mg of graphene oxide and 10 mL of ginsenoside Rh2 were poured into a 100 mL round-bottomed flask, and carefully poured 1 mL of concentrated sulfuric acid down the walls of the flask. Then, the reactants were mixed, and the mixture was heated at reflux with a thermowell for 3 hours at 80 °C. Then, the reaction mixture was cooled to room temperature and subsequently poured it into 75 mL of water and centrifuged. After centrifuging 10 times with water, the supernatant’s pH was adjusted to neutral. Note that the yield of the esterification was increased by using high temperature and removing water by evaporation to shift the equilibrium to the right (Le Chatelier’s principle)^41^.

$${\rm{R}} \mbox{-} {\rm{COOH}}({\rm{Grapheneoxide}})+{\rm{R}}^{\prime} {\rm{OH}}({\rm{GinsenosideRh2}}){\boldsymbol{\to }}{{\rm{RCO}}}_{2}\, \mbox{-}  \mbox{-} \,R^{\prime} +{{\rm{H}}}_{2}{\rm{O}}$$

### Preparation of arginine-treated graphene (Gr-Arg) and lysine-treated graphene (Gr-Lys)

The pristine graphite (10 mg) and 185.4 mg of AlCl_3_ as a Lewis acid were ball-milled and poured into a Teflon reaction vessel, and 200 ml of tetrahydrofurfuryl polyethylene glycol (PEG) were gradually added during sonication for 30 min at room temperature to obtain a homogeneous suspension under the nitrogen atmosphere. A small white smoke was seen while adding the PEG. Also, concentrated hydrochloric acid (0.5 mL) was added dropwise to the graphite suspension during the sonication process. Then, the mixture was poured into a Teflon vessel, sealed and transferred into an industrial microwave and irradiated at 150 °C at an output power of 700 W for 20 min (with frequently of 5 min sonication and 5 min placed under microwave irradiation). An electrophilic addition reaction occurred between the tetrahydrofurfuryl polyethylene glycol and the graphite, resulting in the attachment of the functional groups and hydroxyl groups to the exposed edges and sides of the graphite flakes, thereby producing functionalized, expanded graphite. Microwave irradiation was used to increase the speed of the reaction and the functionalization yield. Subsequently, the resulting black ink-like dispersion was left for 24 h to separate large unstable graphite aggregates. The functionalized graphite, without unstable graphite aggregates, was expanded and was much more soluble in ethylene glycol than the pristine graphite. Then, the homogeneous suspension of PEG-treated expanded graphite in ethylene glycol was poured into another vessel that contained 450 ml of ethylene glycol. The mixture was sonicated for 2 h to completely disperse the functionalized graphite flakes; this was followed by 30 min of centrifugation at 3000 rpm to collect the supernatant, which was filtered, washed, and dried. To obtain pure graphene without functional groups, thermal treatment up to 500 °C under a nitrogen atmosphere was applied for 15 min to remove all functional groups. The product was pure graphene. This procedure was reported by Amiri *et al*.^42^.

Then, to prepare functionalized graphene with Arginine (Gr-Arg), the following procedure was used. The mechanism for functionalization of pure graphene with Arginine includes the generation of semi-stable diazonium ions, which then initiate a radical reaction with the sheets^43^. The initial used amount of Arginine was 50 mg. To this end, pure graphene and deionized water were poured into a bottle. The reaction bottle was sonicated after adding arginine and isoamyl nitrite and concentrated HCl. The resulting black mixture was again poured into a Teflon vessel for microwave irradiation at 700 W for 30 min (with frequency of 5 min sonication and 5 min placed under microwave irradiation). Subsequently, the resulting suspension was mixed vigorously for 36 h, and after cooling to room temperature, it was filtered and washed thoroughly with water, followed by drying for 48 h at 50 °C. The high-dispersibility product, due the functional group of arginine, was sonicated for 4 h in DI water. The resulting black ink-like dispersion with given weight fraction was prepared. The same procedure was performed for the functionalization of pure graphene with Lysine (Gr-Lys).

The same amount of each amino acid was used initially for the synthesis experiments to avoid any errors. The amount of Arg or Lys bound on carbon-based materials was determined by measuring the initial and final concentration of amino acids in the reaction media. Accordingly, the amount of Arg or Lys in the supernatant was determined by HPLC using the standard amino acids solutions. The amount of bound amino acids onto carbon-based nanocomposites was calculated by the following equation.

$${\rm{Bound}}\,{\rm{amino}}\,{\rm{acids}}={\rm{Ci}}-{\rm{Cs}}$$

where Ci and Cs are the concentrations of amino acids initially used for reaction, and the unbound amino acids collected in each purification cycle, respectively.

### Preparation of ginsenoside Rh2-treated Gr-Arg (Gr-Arg-Rh2) and ginsenoside Rh2 -treated Gr-Lys (Gr-Lys-Rh2)

To synthesize ginsenoside Rh2-treated Gr-Arg (Gr-Arg-Rh2), 10 mg of Gr-Arg and 10 mL of ginsenoside Rh2 were poured into a 100 mL round bottomed-flask, and 1 mL of concentrated sulfuric acid carefully poured down the walls of the flask. Then, the reactants were mixed and the mixture was heated at reflux with a thermowell for 3 h at 80 °C. Then, the reaction mixture was cooled to room temperature and subsequently poured into 75 mL of water and centrifuged. After more than 25 times centrifugation with water, the supernatant was tested regarding pH to reach the water pH. Note that the yield of the esterification was increased by using high temperature and removing water by evaporation to shift the equilibrium to the right (Le Chatelier’s principle).

The same procedure was performed for the functionalization of Gr-Lys with ginsenoside Rh2 (Gr-Lys-Rh2).

### Biocompatibility experiments

The *in vitro* biocompatibility experiments of the synthesized nanocarriers were performed in accordance with relevant guidelines and regulations. Red blood cells (RBCs) were provided by healthy volunteers under the approval of Al-Zahra hospital in Isfahan, Iran. Human cell lines were provided from Pasteur Institute of Iran, and were used under the approval of Al-Zahra hospital in Isfahan, Iran. All methods in this section were carried out in accordance with relevant guidelines and regulations, all experimental protocols were approved by Al-Zahra hospital in Isfahan, Iran, and informed consent was obtained from all subjects.

#### Anticancer activity

As a standard colorimetric assay, MTT [3-(4, 5-dimethyl-2-thiazolyl) -2, 5-diphenyl-2H- tetrazolium bromide] was used in this study for the evaluation of cytotoxicity of nanostructures. This assay was done on ovarian cancer (OVCAR3), breast cancer (MDA-MB), human melanoma (A375) and human mesenchymal stem cells (MSCs) cell lines. After 4 h of cell incubation (0.01 × 10^6^) with different concentration of nanostructures (5, 25, 50, 100, 200 and 1000 µg/ml), 20 μL of MTT solution (2.5 mg/mL in phosphate-buffered saline) were added to each well. For each concentration, three wells were considered. The supernatant was removed, and 200 μL of 0.04 M HCl in isopropyl alcohol was added to dissolve the formazan crystals. The optical densities (OD) were evaluated at 570 nm using a Rayleigh spectrophotometer. Nanostructures inhibited the proliferation in more than 50%, were selected for the determination of the half maximal inhibitory concentration (IC_50_). All compounds were tested in three independent experiments. The effect of time on the toxicity of nanostructures was also investigated. To this end, the cell lines were incubated with mentioned concentrations of samples for 24, 48, and 72 h and then, the MTT assay was done.

#### TUNEL assay

The percentage of apoptotic cells in each cell line sample was determined by terminal deoxynucleotidyl transferase-mediated dUTP nick-end labeling (TUNEL) assay by *In Situ* Cell Death Detection Kit (Roche Diagnostics GmbH, Mannheim, Germany). In this method, after treatment of cell lines with different concentrations of designed nanostructures, cell cultured wells were fixed with 4% paraformaldehyde in PBS for 15 minutes at room temperature. After washing with PBS, fixed cells in the wells were incubated with 0.3% H_2_O_2_ in methanol for 1 hour to quench endogenous peroxidase activity. The cell permeability was done with 0.1% Triton X-100 (Sigma Aldrich Company, St. Louis, USA) at 4 °C for 5 min, and then incubated with the TUNEL reaction mixture (50 µl) in a humidified chamber and dark room at 37 °C for 1 hour. After washing in PBS, the samples were stained with 50 µl converter-POD at 37 °C for 1 hour. Samples were washed in PBS and exposed to the DAB (3, 3-diaminobenzidine tetrahydrochloride) (Roche Applied Science, Mannheim, Germany) substrate solution for color development in a dark chamber at room temperature for 10 min. Finally, the samples were counted by inverted light microscope under 400× magnifications in each well. For negative controls, instead of the TUNEL reaction mixture, sample cells were incubated with 50 µl of label solution (without terminal transferase)^44,45^.

#### Hemolysis study

For the assessment of hemolytic activity, 5 ml of the human blood from healthy volunteers was collected in a heparinized tube. The blood was centrifuged at 1500 rpm for 5 min. The red blood cells were collected, and plasma was removed. Then, the pellet was washed five times in physiological saline (pH 7.4) before re-centrifuge at 1500 rpm for 5 min. The purified RBCs were diluted ten times in physiological phosphate. Washed blood samples were stored at 4 °C and used for 6 h in hemolysis assay. Spectrophotometric assay was employed for hemolysis assay. In this assay, 10 µl of different concentrations of each sample was added to 90 µl of cell suspension. The resulting mixture was gently vortexed and incubated for 30 min. After centrifugation, the prepared supernatants were diluted with an equal volume of normal saline to measure the absorbance at 567 nm. In this method, untreated cells and cells treated with 1% Triton X-100 were used as negative and positive controls, respectively.

#### Blood coagulation system

Blood Sample preparation: A blood sample was taken from healthy volunteers with normal physical activity. For the preparation of plasma, whole blood was drawn into the anti-coagulated tube. This tube was centrifuged at 3000 rpm for 15 min and then frozen to −20 °C within 30 min of drawing the blood. The plasma was stored at −80 °C until analysis.

Partial Thrombin Time (PTT): Ten microliters of the serial dilution of graphene-based nanostructures were added to 90 µl of human plasma, and all the samples were incubated for 5 min at 37 °C. Then, 100 µL of calcium chloride solution was added, and the sample was placed into the STAGO instrument for clotting time determination. In control experiment, Na_2_SO_4_/0.2 M HAc/NaAc solution was used as a plasma-treated solution. All the experiments were done in triplicate to minimize the amount of uncertainly.

Prothrombin Time (PT): For this evaluation, 10 µl of each diluted graphene-based nanostructures were added to 90 µl of human plasma, and the sample was incubated for 5 min at 37 °C. Subsequently, 200 µL of clotting factors were added to tubes, and the sample was placed into the STAGO instrument for clotting time determination. In control experiment, Na_2_SO_4_/0.2 M HAc/NaAc solution was used as the plasma-treated solution. All the experiments were done in triplicate to minimize the amount of uncertainly.

#### Agglutination Assessment

Blood Sample Selection:Hemagglutination assay was performed using fresh human blood. A peripheral blood sample was acquired from the healthy subject without hemoglobinopathies.

Agglutination Assay: A stock dispersion of human RBC was prepared by dilution with phosphate buffer saline. All nanostructures were suspended in PBS for reaching isotonic values. Frothy microliters of RBC stock suspension was added to 10 μL of each graphene-based dispersions at different concentrations (5, 25, 50, 100, 200 and 1000 µg/ml) and were mixed thoroughly. The mixed solution was poured into 96-well microplates. The inoculation was incubated at 37 °C for 30 min. Then, 20 μL of the mixture blood-based graphene nanostructures were mounted under cover. Visual evaluation was made by at least 20 random images at 200× magnification in a light microscope. The shape and size of the erythrocytes were assessed.

#### Drug incubation with simulated normal and cancerous cells

To evaluate the performance of designed systems, each sample was poured into dialysis pocket and immersed in a suitable solvent (one solvent with acidic pH (5) and another with neutral pH (7)). It was set on a shaker and monitored within 48 hours. In this period of 48 hours, the medium was replaced with fresh solvent. The media were analyzed for nanosystem presence. The amount of released material in the media was assessed and compared by the color change.

## Results and Discussion

### Material characterization

#### Fourier transform infrared spectroscopy (FTIR)

**Figure 1:**
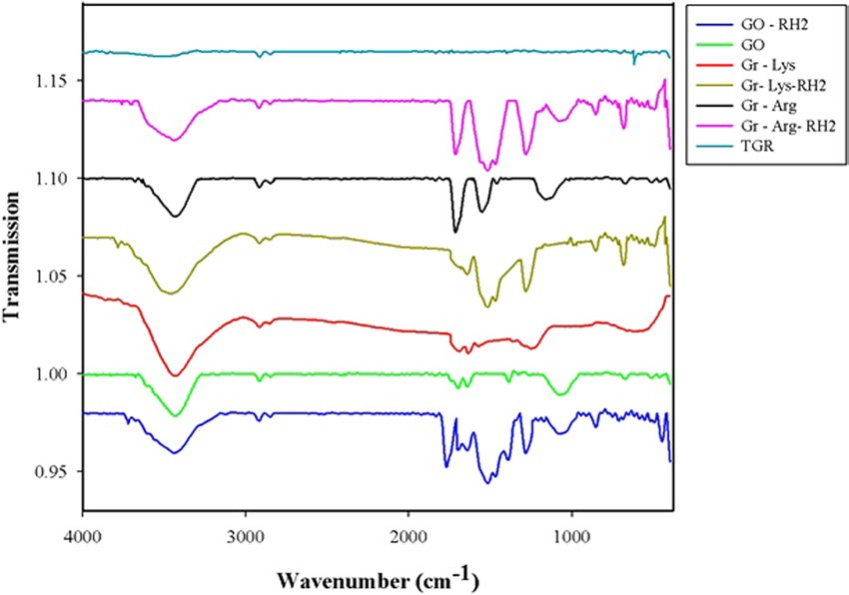
The FTIR spectra of pure graphene, GO, GO-Rh2, Gr-Arg, Gr-Lys, Gr-Arg-Rh2, and Gr-Lys-Rh2.

To study the functional groups, Graphene samples were characterized by infrared spectroscopy (IR), and Raman spectroscopy. Figure 1 shows the FTIR spectra of pure graphene, GO, GO-Rh2, Gr-Arg, Gr-Lys, Gr-Arg-Rh2, and Gr-Lys-Rh2. As shown in this figure, the FTIR spectrum of pure graphene provided no evidence functional groups. The FTIR spectrum of pure graphene showed two weak peaks in the range of 2800–3000 cm^−1^, which were associated with the C-H stretching vibration. Also, the OH stretching vibration produced a weak peak at 3455 cm^−1^, indicating the presence of hydroxyl groups attached to the pure graphene.

In contrast, the FTIR spectrum of all the prepared samples had numerous peaks, confirming the presence of different molecules. The detailed list of peaks and their assigned groups are presented in Table 1. It was observed in the FTIR spectrum of GO that the carboxyl groups had peaks that were considerably sharp. Also, the FTIR spectrum of GO had two peaks in the range of 2800–2950 cm^−1^, which were associated with the C-H stretching vibration. The peaks at 1644 and 1701 cm^−1^ were consistent with COOH and C=O stretching vibrations, respectively, which were infrared-activated by extensive oxidation during hummer procedure. Also, the OH stretching vibration produced a strong and board peak at 3455 cm^−1^, indicating the presence of hydroxyl groups on GO. The FTIR spectrum of the treated samples also had a peak at 1465 cm^−1^, representing the bending vibration of the CH_2_ groups. The peak at 1083 cm^−1^ was in agreement with the stretching vibration of the C–O groups.

**Table 1:** Fourier transforms infrared interpretation of the pure graphene, graphene oxide, graphene oxide-ginsenoside Rh2 (GO-Rh2), arginine-treated graphene (Gr-Arg), lysine-treated graphene (Gr-Lys), ginsenoside Rh2-treated Gr-Arg and ginsenoside Rh2 -treated Gr-Lys.

Samples	Peak(cm^−1^)	Interpretation
Pure graphene	2800–2950	ʋ_s_ (CH_2_) and ʋ_as_ (CH_2_)
	3200–3400	ʋ (C-OH, COOH, H_2_O)
Graphene oxide	1083	ʋ(C–O)
	1366	Vibration modes of epoxide ʋ(C–O–C)
	1644	ʋ (COOH)
	1701	ʋ (C=O)
	2800–2950	ʋ_s_ (CH_2_) and ʋ_as_ (CH_2_)
	3200–3400	ʋ (C-OH, COOH, H_2_O)
Graphene oxide-ginsenoside Rh2 (GO-Rh2)	854	C-H bending (aromatics)
	1047	C-O stretch (alcohols)
	1083	ʋ(C–O)
	1284	C-O-C stretch (diaryl)
	1392	Vibration modes of epoxide ʋ(C–O–C)
	1465	CH_2_ bend
	1517	C=C stretch
	1639	ʋ (COOH)
	1698	ʋ (C=O)
	1770	C=O stretch (esters)
	2800–2950	ʋ_s_ (CH_2_) and ʋ_as_ (CH_2_)
	3200–3400	ʋ (C-OH, COOH, H_2_O)
Arginine-treated graphene (Gr-Arg)	1124	C−O stretching vibration
	1157	−C−N stretching vibration
	1465	−CH_2_ bending vibration
	1552	−NH bending vibration of primary amine
	1714	−C=O stretching vibration
	2800–2950	ʋ_s_ (CH_2_) and ʋ_as_ (CH_2_)
	3300–3600	ʋ (C-OH, COOH, -NH of primary amine, H_2_O)
Lysine-treated graphene (Gr-Lys)	1243	−C−N stretching vibration
	1560	−NH bending vibration of primary amine
	1631	COOH stretching vibration
	1690	−C=O stretching vibration
	2800–2950	ʋ_s_ (CH_2_) and ʋ_as_ (CH_2_)
	3300–3600	ʋ (C-OH, COOH, -NH of primary amine, H_2_O)
**Ginsenoside Rh2-treated Gr-Arg**		
	852	C-H bending (aromatics)
	1045	C-O stretch (alcohols)
	1070	ʋ(C–O)
	1282	C-O-C stretch (diaryl)
	1467	CH_2_ bend
	1514	C=C stretch vibration
	1540	−NH bending vibration of primary amine
	1695	ʋ (C=O)
	1712	C=O stretch (esters)
	2800–2950	ʋ_s_ (CH_2_) and ʋ_as_ (CH_2_)
	3200–3400	ʋ (C-OH, COOH, H_2_O)
**Ginsenoside Rh2 -treated Gr-Lys**		
	848	C-H bending (aromatics)
	1282	C-O-C stretch (diaryl)
	1467	CH_2_ bend
	1515	C=C stretch vibration
	1545	−NH bending vibration of primary amine
	1641	ʋ (COOH)
	1680	ʋ (C=O)
	1702	C=O stretch (esters)
	2800–2950	ʋ_s_ (CH_2_) and ʋ_as_ (CH_2_)
	3200–3400	ʋ (C-OH, COOH, H_2_O)

Besides the above mentioned peaks, it can be seen that the FTIR spectra of GO-Rh2, Gr-Arg- Rh2 and Gr-Lys-Rh2 had some more peaks at 854, 1047, 1284, and 1517 cm^−1^, which were associated with the aromatic C-H bending vibration, C-O stretching vibration from alcohols chains, C-O-C stretching vibration of diaryl, and C=C stretching vibration, respectively. This evidence shows the presence of ginsenoside Rh2 in the samples. Also, The C=O stretching vibration for esters groups with a strong peak at around 1700 cm^−1^ that were observed on the GO-Rh2, Gr-Arg- Rh2 and Gr-Lys-Rh2 samples could indicate that esterification reactions occurred between the carboxyl groups of arginine/lysine or the carboxyl groups on GO surface and the hydroxyl groups of ginsenoside Rh2.

From Gr-Arg and Gr-Lys spectra, decorated amino acids on graphene surface show peaks at ~1200 and 1560 cm^−1^, representing the stretching vibration −C−N and −NH bending vibration of primary amine, respectively. All of the peaks presented in the FTIR spectra of different samples confirmed the successful functionalization of the Gr and GO with the target molecules.

#### Raman Spectroscopy

**Figure 2:**
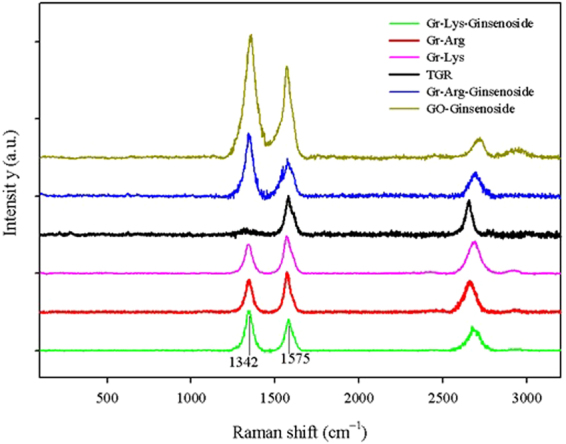
The Raman spectra of the pure graphene, GO, GO-Rh2, Gr-Arg, Gr-Lys, Gr-Arg-Rh2, and Gr-Lys-Rh2.

The Raman spectra of the pure graphene, GO, GO-Rh2, Gr-Arg, Gr-Lys, Gr-Arg-Rh2, and Gr-Lys-Rh2 are shown in Fig. 2. Raman characterization is a strong candidate for analyzing the structure and sp^2^ and sp^3^ hybridized carbon atoms in carbon-based nanomaterials, functionalization and exfoliation by following alterations in hole and electron doping^46,47^. As could be seen in Fig. 2, the Raman spectra of all samples illustrate the D and G bands at around 1342 and 1575 cm^−1^, respectively. The ratio of the D- to G-band intensities (I_D_/I_G_) can be the best criterion for the amount of sp^3^-hybridized carbon relative to sp^2^-hybridized carbon. It is well known that the amount of I_D_/I_G_ means the higher disruption of aromatic π-π electrons in carbon-based nanomaterials, implying the partial damage of graphitic carbon^48^. As could be seen, while the pure graphene illustrates no sharp D band, Gr-Arg, Gr-Lys, Gr-Arg-Rh2 and Gr-Lys-Rh2 samples are shown strong D bands and higher I_D_/I_G_ ratios, implying structural deformation by functionalization. The ratio of the I_D_/I_G_ for all the samples is listed in Table 2. It is noteworthy that the I_D_/I_G_ value of Gr-Arg-Rh2 was larger than that of Gr-Arg. Also, the I_D_/I_G_ value of Gr-Lys-Rh2 was larger than that of Gr-Lys. The higher I_D_/I_G_ of Gr-Arg-Rh2 and Gr-Lys-Rh2 show that the esterification reaction between the carboxyl group of Gr-Arg (or Gr-Lys) and the hydroxyl group of ginsenoside Rh2 was successful.

**Table 2:** The ratio of the I_D_/I_G_ for the pure graphene, GO, GO-Rh2, Gr-Arg, Gr-Lys, Gr-Arg-Rh2 and Gr-Lys-Rh2.

Sample	Amount of I_D_/I_G_
GO	0.598298
GO-Rh2	1.341838
Pure graphene	0.140674
Gr-Arg	0.812139
Gr-Arg-Rh2	1.666906
Gr-Lys	0.777368
Gr-Lys-Rh2	1.268521

The higher I_D_/I_G_ of Gr-Arg-Rh_2_ and Gr-Lys-Rh_2_ show that the esterification reaction between carboxyl group of Gr-Arg (or Gr-Lys) and hydroxyl group of ginsenoside Rh_2_ was successful.

#### High-resolution transmission electron microscopy (HRTEM)

**Figure 3:**
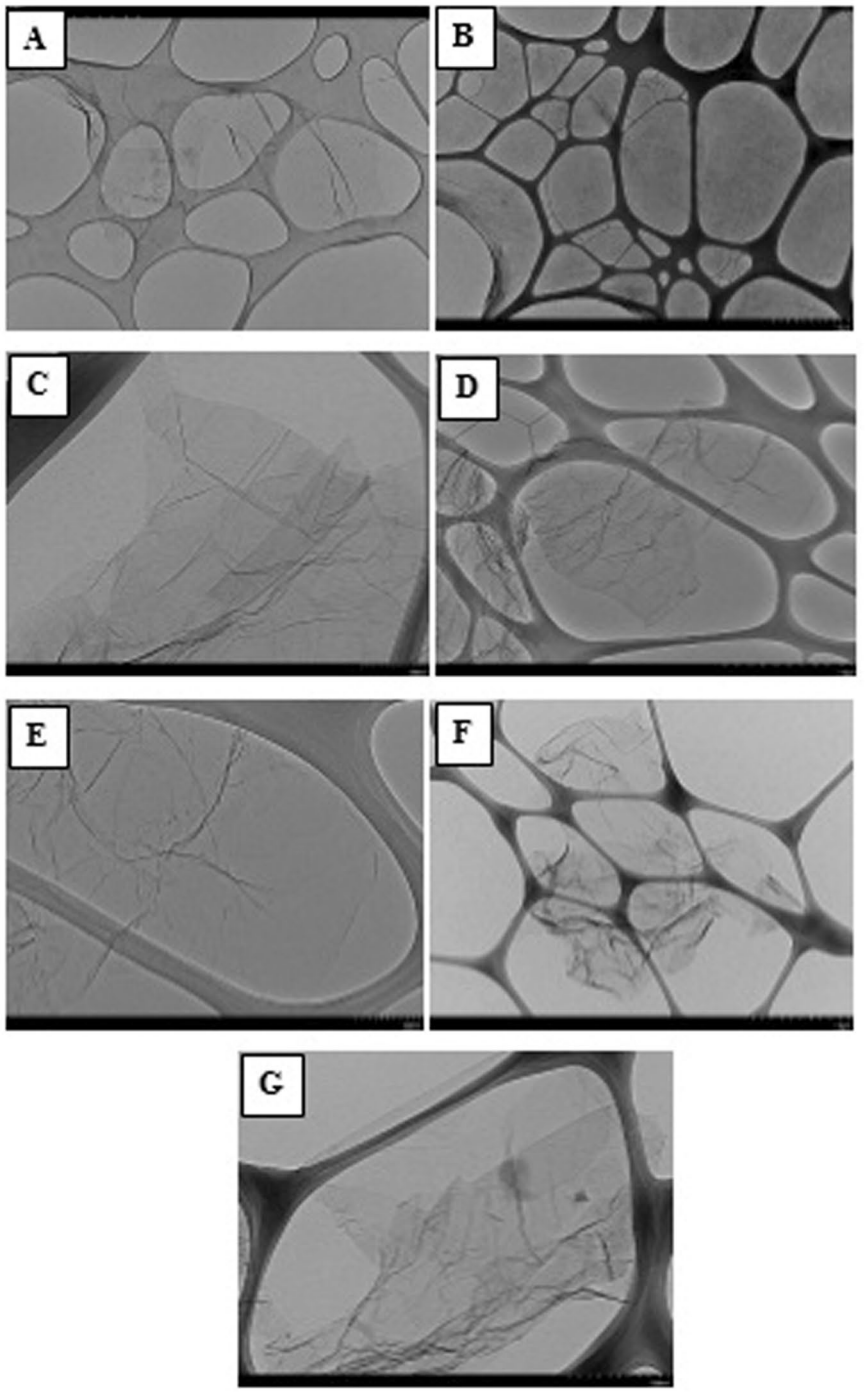
The transmission electron microscopy (TEM) images of the (**A**) pure graphene, (**B**) GO, (**C**) GO-Rh2, (**D**) Gr-Lys, (**E**) Gr-Arg, (**F**) Gr-Lys-Rh2, and (**G**) Gr-Arg-Rh2.

Figure 3 shows the transmission electron microscopy (TEM) images of the pure graphene, GO, GO-Rh2, Gr-Arg, Gr-Lys, Gr-Arg-Rh2, and Gr-Lys-Rh2. All HRTEM images demonstrate some few-layered graphene or GO flakes with diameter around 2 µm. Note that the images of the pure graphene and GO samples show some sheets with more or less smooth layer’s surface. Although HRTEM image is not able to distinguish very small functional groups, surface change, deterioration and the presence of wrinkles in sheets can be considered as a reason for the attachment of functional groups. From the HRTEM images of the Gr-Arg, Gr-Lys, Gr-Arg-Rh2, and Gr-Lys-Rh2, the resulting sheets preserved their shape and size as compared with the pure graphene. Figure 3 also shows the images of the GO-Rh2, Gr-Arg, Gr-Lys, Gr-Arg-Rh2, and Gr-Lys-Rh2 samples, which was comprised of a few-layered graphene with wrinkled morphology and folded edges. Note that the presence of so many lines and wrinkles within the GO-Rh2, Gr-Arg, Gr-Lys, Gr-Arg-Rh2 and Gr-Lys-Rh2 sheets are attributed to the inherent instability of the graphene structures and the enhanced flexibility of sheets after chemical functionalization. The higher tendency for wrinkling shows a growth in the wettability of graphene surface due to covalent functionalization with amino acids and ginsenoside Rh2 as well. The easily-miscible amino acids (Arg and Lys) and ginsenoside Rh2 functionalities can explain the enhanced wettability of the functionalized samples. Consequently, higher dispersion stability in aqueous media for all the samples was obtained. Moreover, a set of highly-crumpled, individual graphene flakes with suitable transparency and without observable graphite crystalline structure in Fig. 3 confirmed that these wrinkles resulted from the crumpling of graphene rather than stacking.

### Cell viability

#### MTT Assay

The cell lines were treated with different concentrations of GO, Rh2, GO-Rh2, Gr-Arg, Gr-Lys, Gr-Arg-Rh2, and Gr-Lys-Rh2 for 24 h. The effect of these types of compounds on cell viability was evaluated by MTT assay. The IC_50_ values of the GO, Rh2, GO-Rh2, Gr-Arg, Gr-Lys, Gr-Arg-Rh2, and Gr-Lys-Rh2 against ovarian cancer (OVCAR3), breast cancer (MDA-MB), Human melanoma (A375) and human mesenchymal stem cells (MSCs) cell lines were presented in Table 3. Overally, A375 cells were more vulnerable to all experiment nanocompounds than the other cell lines. According to the results, Gr-Arg-Rh2 had the highest anticancer activity among all the graphene-based samples. The sequence of anticancer activity of Gr-Arg, Gr-Lys, Gr-Arg-Rh2, and Gr-Lys-Rh2 was A375 > OVCAR3 > MSCs > MDA-MB. Also, this sequence for GO and GO-Rh2 was A375 > MSCs > OVCAR3 > MDA-MB and for Rh2 was OVCAR3 > A375 > MDA-MB > MSCs. There were significant differences between IC_50_ of Gr-Arg-Rh2 and GO, Rh2, Gr-Arg, and Gr-Lys (P value = 0.012). However, differences among IC_50_ of Gr-Arg-Rh2, GO-Rh2, and Gr-Lys-Rh2 were not statistically significant (P value > 0.05). Interestingly, Gr-Arg, Gr-Lys, Gr-Arg-Rh2, and Gr-Lys-Rh2 were more active against cancer cell lines in comparison to their cytotoxic activities against normal cell lines (MSCs) with IC_50_ values higher than 100 μg/ml. Similar results were not repeated in the presence of GO and GO-Rh2. Noteworthy, Gr-Arg-Rh2 and Gr-Lys-Rh2 revealed the highest amount cytotoxicity against all the cell lines as compared with other graphene-based samples. The presence of –NH_2_ groups in the main structures of Arg and Lys impose the positive charge on the main surface of graphene. On the other hand, due to high glycolysis in cancerous cells, the negative charge increased as the lactic acid accumulated, resulting in a decrease in pH. It can be concluded that the increased anticancer ability of the Gr-Lys and Gr-Arg may be related to an electrostatic interaction between the cell membranes and positively charged graphene surface. On the other hand, the anticancer activity of Gr-Arg and Gr-Arg-Rh2 were about twice as other cationic samples like Gr-Lys and Gr-Lys-Rh2. Therefore, the higher activities of Gr-Arg and Gr-Arg-Rh2 are attributed to the higher electrostatic adsorption as compared to Gr-Lys and Gr-Lys-Rh2. Furthermore, the simultaneous presence of two anticancer agents of Rh2 and GO lead to synergism effects and resulted in increased cytotoxicity as well as broader activity spectrum. Previous studies have proven that Rh2 kill cancerous cells with inhibition of cell growth and Angiogenesis as well as induction of apoptosis^49–51^. Thus, it may be concluded that the presence of Rh2 leads to an increase in antitumor activity. Also, the presence of Arg and Lys lead to stronger electrostatic absorption, orientation, and interaction of Gr sheets with cancerous cells. The cytotoxicity of the studied compounds was also evaluated in normal cells. These cell lines were used as the predictive model. As an example, Zhou *et al*. showed that the GO could induce or inhibit the expression of 2254 cellular proteins in breast cancer cells (MDA-MB-231). They indicated that 168 proteins were found to be up-regulated and 685 proteins were down-regulated by GO. GO induced protein expression was involved in metabolic processes and it influenced apoptosis^52^. The decrease of ATP generation, down-regulation of proteins related to energy metabolism, and expression of miRNA are different proposed mechanisms for anticancer activity of GO^52,53^. All the proposed mechanisms are suggested for observed anticancer activities of our designed Gr-based nanostructures. The electrostatic interaction between positively charged nanomaterials and the negative charge of some biological surfaces can be a reason for enhanced biological activity, in particular, anticancer activity of different Gr-based samples as compared to the pure ones^54–56^. GO have a lot of COOH groups, leading a reduction in hydrophobic interaction between nanostructures and healthy mammalian cells. So, the toxicity of functionalized graphene was reduced in comparison with pure graphene. According to our results, the functionalization of Gr with Arg, Lys, and Rh2 changed the effect on the cell lines. This functionalization with the natural groups can reduce the toxicity of pure graphene as well as their size. As an example, Akhavan *et al*. concluded that GO nanoplatelets with the larger size have lower toxicity on MSCs than the smaller size ones^57^. So, increases in size and charge can change the effects of GO on cancerous and normal cell lines. This might indicate the chemical contamination for lineage cells and could alter the normal biological behavior *in vitro*. Ultra-structure, gene expression profiles and *in vivo* study of these compounds might be required to show other features of their effects on live normal and tumor cell lines to describe cell and molecular mechanisms of their functions.

**Table 3:** Cytotoxicity (IC_50_ values) of GO, Rh2, GO-Rh2, Gr-Arg, Gr-Lys, Gr-Arg-Rh2, and Gr-Lys-Rh2 against different cell lines.

IC_50_ (µg/ml)				
Compounds	MSCs	A-375	OVCAR3	MDA-MB
GO	70.83	56.76	126.47	146.12
Rh2	173.54	123.8	98.82	140.06
GO-Rh2	59.57	48.39	110.71	120.91
Gr-Arg	131.49	57.44	125.08	136.05
Gr-Lys	129.71	60.03	127.42	132.89
Gr-Arg-Rh2	119.02	30.37	72.13	125.96
Gr-Lys-Rh2	123.59	32.08	76.187	130.96

Nanostructures inhibited the proliferation in more than 50% were selected for the determination of the half maximal inhibitory concentration (IC_50_).

#### TUNEL assay

**Figure 4:**
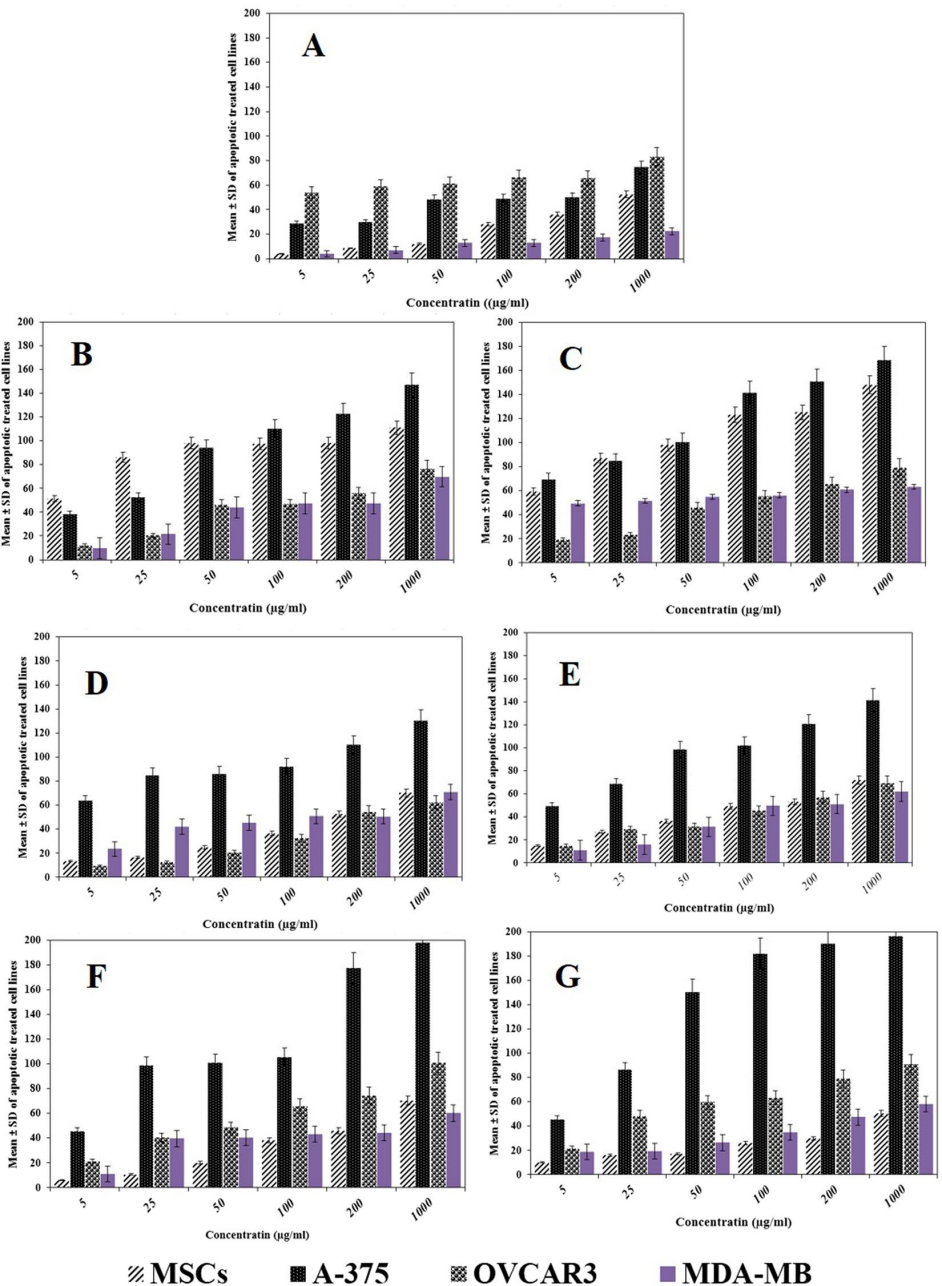
The results of TUNEL assay after incubation of cells with different concentrations of nanostructures. (**A**) Rh2, (**B**) GO, (**C**) GO-Rh2, (**D**) Gr-Lys, Gr-Arg, (**F**) Gr-Lys-Rh2, and (**G**) Gr-Arg-Rh2.

One of the most important methods for detection of DNA fragmentation and chromatin quality is Terminal deoxynucleotidyl transferase dUTP nick end labeling (TUNEL). This method is done by labeling the 3′-hydroxyl terminal in the double-strand DNA breaks generated during apoptosis. So, this method is very suitable for detection of apoptotic DNA fragmentation, widely used to identify and quantify apoptotic cells^58,59^. The results of TUNEL assay were summarized in Fig. 4 and Table 4. Acquired data indicates a significant increase in the rates of TUNEL positive cells based on increased the concentrations of materials compared to control groups that was in consistent with the findings of MTT assay. The results showed that the highest toxicity of nanostructures belonged to Gr-Lys-Rh2 on A375 cell line similar to MTT assay results. The highest significant difference was observed among Go, Gr-Lys-Rh2, and Gr-Arg-Rh2 for normal and cancerous cell lines, between Go and Rh2 for normal cell lines as well as among Rh2, Gr-Lys-Rh2, and Gr-Arg-Rh2 for A-375 cell lines.

**Table 4:** Comparison of Mean of apoptotic treated cell lines with different concentrations of nanomaterials.

Variables		P value						
Cell lines	Nanostructures	Rh2	Go	GO-Rh2	Gr-Lys	Gr-Arg	Gr-Lys-Rh2	Gr-Arg-Rh2
MSCs	Rh2	—	0.000	0.012	0.09	0.085	0.112	0.122
	Go	0.000	—	0.235	0.06	0.071	0.045	0.041
	GO-Rh2	0.012	0.235	—	0.063	0.068	0.036	0.04
	Gr-Lys	0.09	0.06	0.063	—	0.562	0.053	0.048
	Gr-Arg	0.085	0.071	0.068	0.562	—	0.046	0.041
	Gr-Lys-Rh2	0.112	0.045	0.036	0.053	0.043	—	0.812
	Gr-Arg-Rh2	0.122	0.041	0.04	0.048	0.041	0.812	—
MDA-MB	Rh2	—	0.352	0.045	0.125	0.10	0.09	0.068
	Go	0.352	—	0.037	0.056	0.061	0.041	0.035
	GO-Rh2	0.045	0.037	—	0.051	0.065	0.09	0.07
	Gr-Lys	0.125	0.056	0.051	—	0.435	0.075	0.085
	Gr-Arg	0.10	0.061	0.065	0.435	—	0.091	0.125
	Gr-Lys-Rh2	0.09	0.041	0.09	0.075	0.091	—	0.251
	Gr-Arg-Rh2	0.068	0.035	0.07	0.085	0.125	0.251	—
A-375	Rh2	—	0.032	0.026	0.045	0.048	0.020	0.034
	Go	0.032	—	0.074	0.145	0.156	0.036	0.028
	GO-Rh2	0.026	0.074	—	0.047	0.051	0.047	0.045
	Gr-Lys	0.045	0.156	0.051	—	0.514	0.025	0.031
	Gr-Arg	0.048	0.156	0.051	0.514	—	0.05	0.049
	Gr-Lys-Rh2	0.020	0.036	0.047	0.025	0.05	—	0.652
	Gr-Arg-Rh2	0.034	0.028	0.045	0.031	0.049	0.652	—
OVCAR3	Rh2	—	0.054	0.062	0.074	0.124	0.051	0.067
	Go	0.054	—	0.089	0.155	0.210	0.039	0.041
	GO-Rh2	0.062	0.089	—	0.023	0.021	0.042	0.038
	Gr-Lys	0.074	0.155	0.023	—	0.842	0.051	0.029
	Gr-Arg	0.124	0.210	0.215	0.842	—	0.039	0.041
	Gr-Lys-Rh2	0.051	0.039	0.042	0.051	0.039	—	0.726
	Gr-Arg-Rh2	0.067	0.041	0.038	0.029	0.041	0.726	—

Note: P values of less than 0.05 were regarded as statistically significant.

### Hemolysis assay and morphology of RBCs

**Figure 5:**
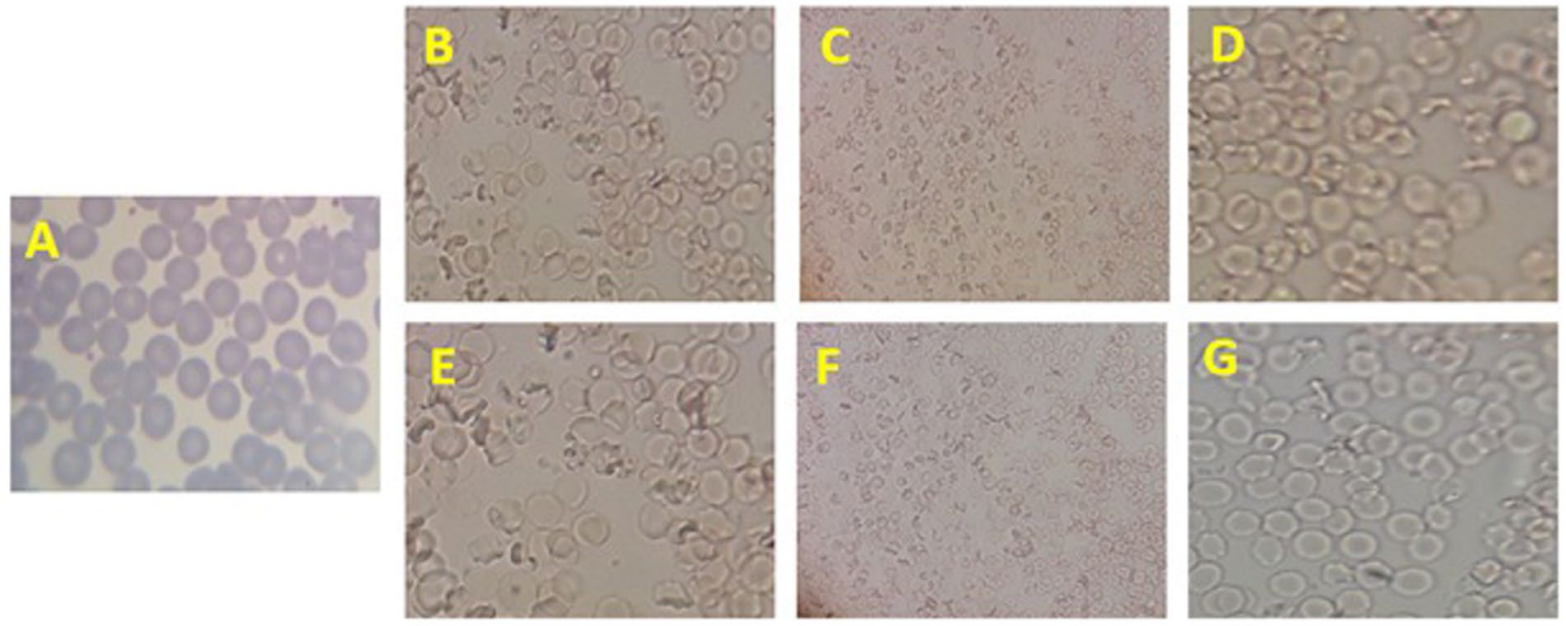
Changes of RBCs morphology in the presence of GO, GO-Rh2, Gr-Arg, Gr-Lys, Gr-Arg-Rh2, and Gr-Lys-Rh2. (**A**) Rh2 1 mg/ml, (**B**) GO 200 µg/ml, (**C**) GO-Rh2 200 µg/ml, (**D**) Gr-Lys 400 µg/ml, (**E**) Gr-Arg 400 µg/ml, (**F**) Gr-Lys-Rh2 400 µg/ml, and (**G**) Gr-Arg-Rh2 400 µg/ml. The minimum concentration of each nanostructure that leads to change the RBCs morphology was shown in this figure.

Hemolytic assays were done because nanomaterials with potent biological activity may not be useful in pharmacological preparations if they have hemolytic effect. *In vitro* hemolytic activity on human erythrocytes of various concentrations of GO, Rh2, GO-Rh2, Gr-Arg, Gr-Lys, Gr-Arg-Rh2, and Gr-Lys-Rh2 was performed and the results were presented in Table 5. As shown in this table, the GO exhibited the greatest hemolytic activity than Rh2, Gr-Arg-Rh2, and Gr-Lys-Rh2 nanocomposites. However, all the graphene-based nanostructures had hemolytic effect on erythrocytes. Upon keeping the same conditions, the hemolytic activity of all examined nanostructures increased with concentration. Rh2 showed no hemolytic activity in all concentrations. A maximum 50% hemolysis occurred in the presence 250 μg/ml, 360 μg/ml, 420 μg/ml, 435 μg/ml, 500 μg/ml, and 575 μg/ml of GO, GO-Rh2, Gr-Lys, Gr-Arg, Gr-Lys-Rh2, and Gr-Arg-Rh2, respectively. As obvious in this table, all nanostructures proved to be hemolytic. Note that Rh2, Gr-Arg-Rh2, and Gr-Lys-Rh2 produced hemolytic zones on the red background lower than GO after 30 min incubation at all concentrations. Liao *et al*. investigated the effects of graphene on human erythrocytes. They indicated that nano-sized graphene could induce severe hemolysis compared to micro-sized (3 μm) graphene sheets. In our study, to prepare Gr-Arg, Gr-Lys, Gr-Arg-Rh2, and Gr-Lys-Rh2, no acid-treatment was used, resulting in preparation of Gr sheets with larger size than that of GO-based samples^60^. So, reduction of hemolytic activity between examined nanostructures with the sequence of GO > GO-Rh2 > Gr-Lys > Gr-Arg > Gr-Lys-Rh2 > Gr-Arg-Rh2, could be due to increased size of nanostructures. On the other hand, the electrostatic interactions between the graphene surface and the lipid membrane of RBCs lead to serious membrane disruption. Liao *et al*. have also proven that the hemolytic activity and disruption of RBCs membrane is related to electrostatic interactions between negatively charged oxygen groups on the GO surface and positively charged phosphatidylcholine lipids of RBC’s outer membrane^60^. In our study, addition of Lys and Arg increased the positive charge of Gr surface. So it may be caused by a repulsive force between functionalized Gr and RBCs. Thus, the reduction of surface areas by functionalization and increase of size and positive charge reduce the impact of pure Gr on RBCs. The shape of RBCs leads to the maximized uptake of oxygen and carbon dioxide from the surrounding. RBCs with this shape have flexibility and easily flow through the capillaries, even if the diameter is smaller than that of RBCs. External materials may have undesirable interaction with RBCs and induce biosafety issues. The results of this study showed that the aggregation and change of RBCs morphology occurred in the presence of GO, GO-Rh2, Gr-Arg, Gr-Lys, Gr-Arg-Rh2, and Gr-Lys-Rh2. Microscopic studies showed in turn the grid surface of RBCs after incubation with graphene (Fig. 5). These changes were significant at 200 µg/ml or more for GO and GO-Rh2 and 400 µg/ml for Gr-Arg, Gr-Lys, Gr-Arg-Rh2, and Gr-Lys-Rh2. At 5–100 µg/ml, none of nanostructures cause RBC aggregation and morphological change. GO lead to RBC aggregation and morphological change in lower concentration than other nanostructures (25 µg/ml). So, all of nanostructures obtained a clear tendency to aggregate which was evidenced by an increase in the concentration, but functionalized graphene had lower aggregation activity. The data was compatible with other studies that showed the hemolytic activity of Gr sheets and relationship between size, charge and aggregation in this nanostructures^60–64^. Enhancement of positive charge by addition of Lys and Arg led to a reduction in the effect on RBCs membrane and morphology.

**Table 5:** The hemolytic activity of GO, Rh2, GO-Rh2, Gr-Arg, Gr-Lys, Gr-Arg-Rh2, and Gr-Lys-Rh2 on human RBCs.

	Rh2	GO	GO-Rh2	Gr-Lys	Gr-Arg	Gr-Lys-Rh2	Gr-Arg-Rh2
50% hemolysis	—	250	360	420	435	500	575

The absorbanse of Triton X-100 (1%), which yields full hemolysis, was considered as 100% hemolysis and the absorbacne of other treated RBCs by nanostructures were compared with the Triton X-100 by this frormula: %hemolysis = OD _(test)_ − OD _(balnk)_/OD _(triton X-100)_ − OD _(blank)_ × 100. After this, plot the percentage lysis was drawn based on absorbance. The structures required for 50% haemolysis for the control and test serum was calculated.

### Coagulation assay

The coagulation test is a common method for hemocompatibility evaluation of biomaterials. In assessment of blood coagulation, intrinsic (PTT) and extrinsic (PT) pathways were evaluated^65,66^. The results of this section were shown in Table 6. As shown in this table, an increase in PT and PTT was observed as the concentration of GO, GO-Rh2, Gr-Arg, Gr-Lys, Gr-Arg-Rh2, and Gr-Lys-Rh2 increased. However the rates of enhancements were insignificant in all concentrations of PT and concentrations lower than 100 µg/ml for PTT. An increase in PTT was higher than that of PT. According to similar study, this result may be due to interaction with the intrinsic coagulation factors^63^. One of the most important things about coagulation is the idea of the intrinsic and extrinsic arms of the coagulation cascade, and the way they interact during coagulation in the body. For potent application of any compound, investigation of its effect on blood coagulation is necessary. According to the results, all of the graphene-based samples had effect on blood coagulation system, especially on PTT. Thus, it can be concluded that Gr-based samples have more effect on intrinsic than extrinsic coagulation because of interaction with the coagulation factors. Extrinsic pathway of blood coagulation was evaluated to monitor patients taking certain medications as well as to help diagnose clotting disorders^67,68^. The prothrombin test specifically evaluates the presence of prothrombin. A prothrombin time for 11–15 seconds indicates the normal amounts of the clotting factors. So, this test evaluate prothrombin activity^69^. There is no significant prothrombin activity for all examineed nanostructures except GO. The higher concentrations of GO led to slight reduction of PT. These results indicated that functionalized Gr sheets do not influence the extrinsic pathway of blood coagulation. PTT measures the integrity of the intrinsic systems (Factors XII, XI, VIII, IX) and common clotting pathways. Increased levels in a person with a bleeding disorder indicate the missing of clotting factors. Note that functionalized Gr samples with 100 µg/ml or more concentrations had significant effect on PTT. The effect of all nanostructures on PTT was more than the effects on PT. The effect of all nanostructures was not significant on PTT in concentration lower than 100 µg/ml.

**Table 6:** The effect of GO, Rh2, GO-Rh2, Gr-Arg, Gr-Lys, Gr-Arg-Rh2, and Gr-Lys-Rh2 on Prothrombin Time (PT) and Partial Thromboplastin Time (PTT).

**PT (Sec)**						
Sample (µg/ml)	5	25	50	100	200	1000
Control	13.3	13.3	13.3	13.3	13.3	13.3
Rh2	13.3	13.4	13.3	13.5	13.4	13.4
GO	13.5	13.9	13.5	14	14	13.9
GO-Rh2	13.3	13.3	13.4	13.3	13.8	13.8
Gr-Lys	13.5	13.4	13.3	13.7	13.4	13.5
Gr-Arg	13.3	13.3	13.6	13.5	13.4	13.3
Gr- Lys -Rh2	13.3	13.3	13.3	13.4	13.4	13.5
Gr-Arg-Rh2	13.4	13.4	13.3	13.5	13.3	13.4
**PTT (Sec)**						
Sample (µg/ml)	5	25	50	100	200	1000
Control	34	34	34	34	34	34
Rh2	34	34	34	34	34	34
GO	45	47	48	65	65	70
GO-Rh2	40	45	45	60	64	65
Gr-Lys	38	45	46	58	62	68
Gr-Arg	40	43	45	60	60	67
Gr- Lys -Rh2	36	46	46	56	61	70
Gr-Arg-Rh2	35	45	45	57	60	63

The modeling of graphene interaction with the membrane has shown that the cytotoxicity of these nanostructures depend on penetration into lipid bilayers. If this penetration is slow, the cytotoxicity will decrease. Jo *et al*. showed that the toxicity of graphene and its derivatives increases along with hydrophobicity. The hydrophobicity of graphene causes the instability of lipid bilayer. This situation leads to instability of blood coagulation proteins on lipid bilayer^70^. Addition of Rh2, Lysine, and Arginine reduce the hydrophobicity of graphene. So, functionalization of graphene with these compounds led to the reduction of the effects on blood coagulation in comparison with GO.

### *In vitro* assessment of nanosystems

**Figure 6:**
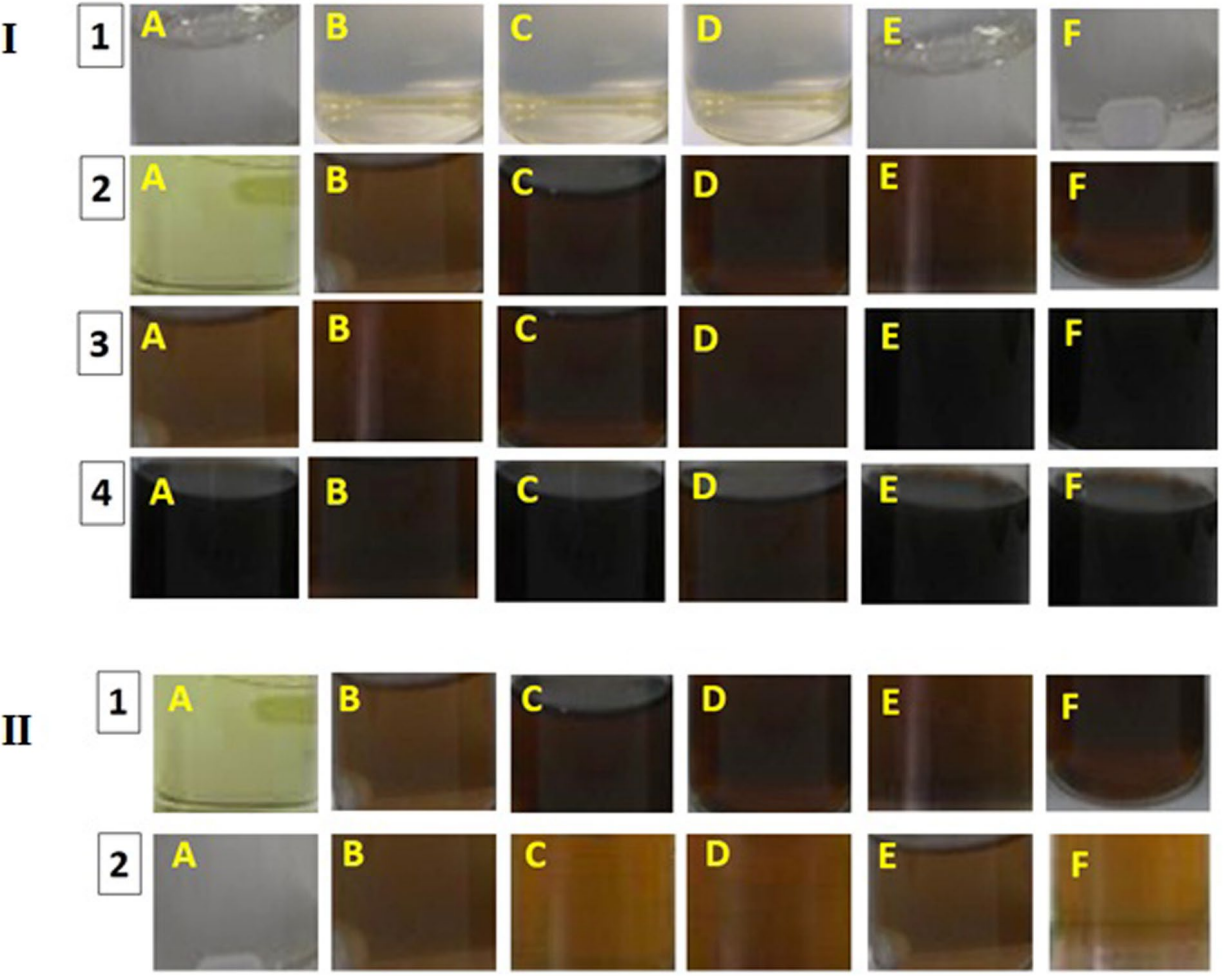
*In vitro* assessment of nanosystems by their incubation in dialysis pocket. (**I**): The analyzed for the presence of nanosystem in the medium after 0 h (1), 6 h (2), 12 h (3), and 24 h (4) incubation in acidic pH. (**II**): Comparison of the presence of nanosystem in acidic (1) and neutral (2) after 12 h incubation. (A) GO-Rh2, (B) GO, (C) Gr-Lys, (D) Gr-Arg, (E) Gr-Lys-Rh2, and (F) Gr-Arg-Rh2.

The analysis of medium around dialysis pocket after incubation showed that all nanostructures have synergism effect with Rh2. All structures act as an antitumor drugs such that binding of drugs to a nanostructure is irresolvable and the whole structure enter into the cell as an effective drug in both pHs. On the other hand, accumulation rate of GO and GO-Rh2 was similar in the acidic and neutral medium. However, for functionalized nanosystems with Arg and Lys, this accumulation rate was faster and more efficient in the acidic medium than in the neutral one. This means that the release of nanosystems is faster at acidic pH. As is shown in Fig. 6, Gr-Arg, Gr-Arg-Rh2, Gr-Lys, and Gr-Lys-Rh2 have more suitable conditions than other samples. This might be due to the higher positive charges of these nanosystems (due to the presence of charged amino acids). This status leads to increased electrostatic interaction. Cancerous cells have acidic media due to high glycolysis and fermentation^71–73^. So, the increased ability of functionalized Gr with Lys and Arg to electrostatic interaction with cancerous cells can increase the tendency of functionalized nanostructures to these cells. Thus, positively-charged arginine and lysine groups of functionalized Gr may act as electrostatic adsorbents of the negatively-charged membranes of cancerous cells. So, it may be concluded that the higher membrane adsorption by charged functionalities leads to increased interaction of Gr-Arg, Gr-Arg-Rh2, Gr-Lys, and Gr-Lys-Rh2 with the membranes of cancerous cells. As a result, the Gr-Arg, Gr-Arg-Rh2, Gr-Lys, and Gr-Lys-Rh2 may penetrate the cell with more efficiency.

## Conclusion

In this study, Rh2–treated graphene oxide (GO-Rh2), lysine-treated highly porous graphene (Gr-Lys), arginine-treated Gr (Gr-Arg), Rh2–treated Gr-Lys (Gr-Lys-Rh2) and Rh2–treated Gr-Arg (Gr-Arg-Rh2) were synthesized. Interestingly, Gr-Arg, Gr-Lys, Gr-Arg-Rh2, and Gr-Lys-Rh2 were more active against cancer cell lines in comparison with their cytotoxic activity against normal cell lines (MSCs). The side effects of functionalized graphene with Arg and Lys was lower than non-functionalized graphene. So, modification with basic amino acids may be a promising strategy to enhance the therapeutic index for anti-cancer agents such as Rh2 because of reduction of side effects on normal cells, enhancement of their anticancer activity as well as the increase of their stability.
